# Editorial: Chemical Insights Into the Synthetic Chemistry of Quinazolines and Quinazolinones: Recent Advances

**DOI:** 10.3389/fchem.2020.641321

**Published:** 2021-02-04

**Authors:** Imtiaz Khan, Yongmin Ma, Aamer Saeed

**Affiliations:** ^1^ Department of Chemistry and Manchester Institute of Biotechnology, The University of Manchester, Manchester, United Kingdom; ^2^ School of Pharmaceutical and Chemical Engineering, Taizhou University, Taizhou, China; ^3^ Department of Chemistry, Quaid-i-Azam University, Islamabad, Pakistan

**Keywords:** aza-heterocycles, sustainable chemistry, enzyme inhibitors, BVDV inhibitors, medicinal applications

Heterocycles are among the well-established classes of compounds in organic chemistry contributing enormously in the generation of small molecule therapeutics due to their distinct biological importance. Among them, aza-heterocycles are immensely important scaffolds largely due to their multifarious agrochemical and medicinal applications. Nitrogen-containing heterocycles are omnipresent in designed bioactive molecules, natural products, and pharmaceutical agents, and are of particular interest in drug discovery arena as reflected by a high proportion of FDA-approved drugs incorporating at least one *N*-heterocyclic structure. In this context, quinazolines and quinazolinones hold a very prominent position due to their diverse pharmacological profile. The variety of synthetic methods offering a facile, operationally simple, and high yielding access to these motifs raise the interest level of synthetic and medicinal chemists to continually explore the new avenues affording drug-like molecules based on these entities.

In designing this article collection based on high-quality reviews and original research, we have tried to cover both synthetic and medicinal aspects of quinazolines and quinazolinones. The research presented in this collection will summarize the recent developments in green and sustainable methodologies as well as identify new opportunities in the exploration of medicinally relevant drug candidates based on quinazoline and quinazolinone motifs.

Microwave-assisted synthesis has emerged as a powerful tool kit for the formation of organic molecules. The use of microwave irradiation not only provides a facile access to the desired molecules at much faster rate and shorter time but also improves the selectivity of the reaction. Mohammadkhani and Heravi have comprehensively covered the synthetic approaches adopted for the formation of quinazoline and quinazolinone skeletons using microwave irradiation as a greener and sustainable source of energy. Employing a range of easily accessible starting materials, various cyclization reactions such as intramolecular heterocyclization, intramolecular Friedel–Crafts–type cyclization, intramolecular azido-reductive cyclization, palladium-catalyzed annulations, cyclocondensation, and multicomponent reactions were attempted, leading to the formation of quinazoline and quinazolinone derivatives.

Bovine viral diarrhea (BVD) is an economically significant disease of cattle and is found in many countries throughout the world, causing considerable financial losses within the livestock industry. Over the last few decades, several selective anti-BVDV compounds have been reported, including virus-targeting and host-targeting derivatives, for example, polymerase inhibitors, protease inhibitors, human cellular enzymes inhibitors, and entry inhibitors. Fernandez et al., in their original research contribution, underscored the anti-BVDV activity of a new family of aza-heterocyclic compounds based on quinazolines. Using a structure-based virtual screening approach, several new inhibitors with favorable modifications were generated. The molecular docking analysis of the potent inhibitor showed the binding into a pocket located in the fingers and thumb domain in BVDV RdRp (RNA-dependent RNA polymerase). Collectively, the assessment of the antiviral activity results offers newly developed quinazoline molecules as potent hits for optimizing antivirals for BVDV (Bovine viral diarrhea virus).

Faisal and Saeed summarized the recent developments in the synthetic methods for the construction of quinazolines. Various eco-friendly, mild, atom-efficient, and high yielding methodologies have been discussed. A wide range of easily accessible and commercially available starting materials were transformed into quinazolines using catalysts based on transition metals such as ruthenium, rhodium, palladium, gold, cobalt, nickel, iron, and copper. Moreover, metal-free approaches involving heterogeneous catalytic systems, microwave-assisted reactions, multicomponent reactions, ionic liquid-catalyzed reactions, and visible light-mediated synthetic systems producing desired heterocyclic core have also been discussed.

Carbonic anhydrases (CAs, EC 4.2.1.1), zinc-containing metalloenzymes, are well-known therapeutic targets for the treatment of several pathological disorders. These enzymes are involved in important biological and pathophysiological functions, such as tumorigenicity, gluconeogenesis, bone resorption, lipogenesis, calcification, ureagenesis, and electrolyte secretion, as well as carbon dioxide and pH homeostasis. In view of the emerging use of carbonic anhydrase inhibitors for the treatment of diabetic retinopathy (a leading cause of vision loss), Khan et al. synthesized a series of quinazolinone compounds using a facile approach. Based on the biological screening results against bovine carbonic anhydrase-II (*b*CA-II) and human carbonic anhydrase-II (*h*CA-II), structure–activity relationship analysis, kinetics data, and molecular docking analysis, this class of *N*-heterocycles could serve as potential drug candidates.

In summary, the original research and reviews covered in this collection clearly underscored the importance of quinazoline and quinazolinone heterocycles. This continued importance will surely stimulate the development of innovative methods or improvement of existing protocols to explore possible drug candidates with desirable structural/functional properties.

## Author Contributions

All authors listed have made a substantial, direct, and intellectual contribution to the work and approved it for publication.

## Conflict of Interest

The authors declare that the research was conducted in the absence of any commercial or financial relationships that could be construed as a potential conflict of interest.

